# Advances and Challenges of Cannabidiol as an Anti-Seizure Strategy: Preclinical Evidence

**DOI:** 10.3390/ijms232416181

**Published:** 2022-12-19

**Authors:** Cecilia Zavala-Tecuapetla, Hiram Luna-Munguia, María-Leonor López-Meraz, Manola Cuellar-Herrera

**Affiliations:** 1 Laboratory of Physiology of Reticular Formation, National Institute of Neurology and Neurosurgery, Insurgentes Sur 3877, La Fama, Mexico City 14269, Mexico; 2 Departamento de Neurobiologia Conductual y Cognitiva, Instituto de Neurobiologia, Universidad Nacional Autonoma de Mexico, Campus UNAM-Juriquilla, Queretaro 76230, Mexico; 3 Instituto de Investigaciones Cerebrales, Universidad Veracruzana, Luis Castelazo Ayala s/n, Col. Industrial Ánimas, Xalapa 91190, Mexico; 4 Epilepsy Clinic, Hospital General de México Dr. Eduardo Liceaga, Dr. Balmis 148, Doctores, Mexico City 06720, Mexico

**Keywords:** cannabidiol, preclinical models, seizure, epilepsy, drug resistant epilepsy, phytocannabinoids

## Abstract

The use of Cannabis for medicinal purposes has been documented since ancient times, where one of its principal cannabinoids extracted from *Cannabis sativa*, cannabidiol (CBD), has emerged over the last few years as a promising molecule with anti-seizure potential. Here, we present an overview of recent literature pointing out CBD’s pharmacological profile (solubility, metabolism, drug-drug interactions, etc.,), CBD’s interactions with multiple molecular targets as well as advances in preclinical research concerning its anti-seizure effect on both acute seizure models and chronic models of epilepsy. We also highlight the recent attention that has been given to other natural cannabinoids and to synthetic derivatives of CBD as possible compounds with therapeutic anti-seizure potential. All the scientific research reviewed here encourages to continue to investigate the probable therapeutic efficacy of CBD and its related compounds not only in epilepsy but also and specially in drug-resistant epilepsy, since there is a dire need for new and effective drugs to treat this disease.

## 1. Introduction

*Cannabis* is a genus of plants that belongs to the Cannabaceae family [1]. Among its species, C. *sativa* L. is a chemically complex plant due to the at least 545 different chemical compounds identified since the 1940s [2], which include more than 100 phytocannabinoids [3,4].

Being the second most abundant phytocannabinoid and due to its multiple effects resulting from its actions on several molecular targets, cannabidiol (CBD) has gained attention due to its attractive therapeutic potential to treat cancer [5], pain [6], Huntington’s disease [7], several psychiatric disorders [8], epilepsy [9], Alzheimer’s disease [10], Parkinson’s disease [11], autoimmune diseases such as rheumatoid arthritis (RA) [12,13], and lately, COVID-19 [14,15], among others.

Currently, new clinical trials on the efficacy of CBD not only for RA (NCT04911127) but also for COVID-19 (NCT04686539, NCT04467918) and lately also for post-COVID syndrome (Long-COVID, NCT04997395) are being conducted (consult ClinicalTrials.gov).

## 2. Cannabidiol (CBD)

CBD is a highly lipophilic terpenophenol with a wide range of pharmacological effects but without psychoactive consequences [16]. CBD has showed anti-inflammatory [17,18], immunomodulatory [19], and neuroprotective [20] effects. Regarding the latter, CBD's anti-oxidant properties [17,21,22] seems to reduce apoptosis and favors neuroprotective effects and cell viability [23,24], effects that can be therapeutically explored.

Among its pharmacological characteristics, CBD shows poor oral bioavailability (around 13–19%) [25]. Recent evidence points out the existing need to really understand how the co-administration of food and oil content in excipients impacts or not CBD’s pharmacokinetics behavior and dosing schemes, which is crucial information for its therapeutic success and for the development of new innovative formulations [26,27]. Moreover, CBD undergoes hepatic conjugative [28] and oxidative metabolism by specific cytochrome P450 enzymes such as CYP3A4, CYP2C19, CYP1A1, CYP1A2, CYP2C9, CYP2D6, and CYP3A5 [29]. In this regard, the possible interactions between CBD, other drugs, and these enzymes should be considered in order to avoid altering CBD's bioavailability.

Finally, sex differences in CBD's actions should not be ruled out [30,31], and although the idea that CBD has low toxicity has prevailed, recent studies indicate increasing levels of liver enzymes (alanine aminotransferase (ALT); aspartate aminotransferase (AST)) due to CBD treatment [32,33,34]. CBD's effects caused by prolonged use on patients with chronic diseases should be kept in mind, since main adverse effects include: headache, drowsiness, decreased appetite, diarrhea, skin rashes, fatigue, and sleep disorders [9,34].

### 2.1. Cannabidiol: A Multitarget Molecule

**Figure 1:**
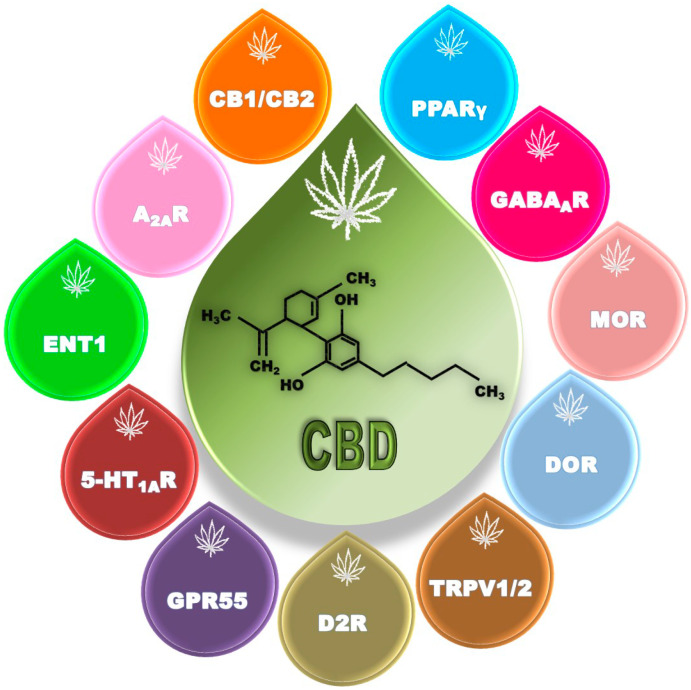
Some molecular targets of CBD’s actions in the brain. CBD acts as a negative allosteric modulator of CB1, MOR, and DOR receptors; as an inverse agonist of CB2 receptors; as an agonist of A_2A_, 5-HT_1A_, D2, PPARү, TRPV1, TRPV2, and GABA_A_ receptors; as an antagonist of GPR55 receptor; and as an inhibitor of ENT1. CBD, cannabidiol; CB1, cannabinoid receptor type 1; CB2, cannabinoid receptor type 2; A_2A_R, adenosine receptor 2A; ENT1, equilibrative nucleoside transporter 1; 5-HT_1A_R, serotonin receptor 1A; D2, D2-like dopamine receptor; GPR55, G-protein-coupled receptor 55; PPARγ, peroxisome proliferator-activated receptor gamma; GABA_A_R, gamma-aminobutyric acid receptor A; MOR, mu opioid receptor; DOR, delta opioid receptor; TRPV1, transient receptor potential vanilloid type 1; TRPV2, transient receptor potential vanilloid type 2.

The pharmacodynamic profile of CBD is complex due to its interaction with a wide variety of molecular targets including enzymes, transporters, receptors, and ion channels, among others (Figure 1) [6,35]. Remarkably, these interactions trigger specific mechanisms and signaling pathways culminating in very diverse effects, with high and broad therapeutic potential for diverse diseases.

#### 2.1.1. Cannabinoid Receptors

Cannabinoid receptors (cannabinoid receptor type 1, CB1; cannabinoid receptor type 2, CB2) activate Gi/o proteins which leads to membrane hyperpolarization [36] and to a decrease in neurotransmitter release from pre-synaptic terminals [37].

CBD has showed a poor affinity for both CB1 and CB2 receptors [38,39]. However, it acts as a non-competitive negative allosteric modulator of CB1 receptors [40,41], while with CB2 receptors, CBD acts as an inverse agonist but only at high concentrations [42]. Furthermore, it has been reported that CBD inhibits the reuptake of anandamide and its degradation by the fatty acid amide hydrolase (FAAH), which increases the endogenous cannabinoid tone; in this way CBD indirectly activates CB1 receptors [42,43,44].

#### 2.1.2. Adenosine Receptors

Adenosine is an endogenous purine nucleoside that regulates several physiological functions via four cell-surface G-protein-coupled receptors (A_1_, A_2A_, A_2B_, and A_3_) [45]. Anti-inflammatory and neuroprotective effects of CBD can be due to either acting as an agonist of A_2A_ receptors, or as a competitive inhibitor at the equilibrative nucleoside transporter 1 (ENT1), which mediates the reuptake of adenosine and therefore its blockade leads to an increase in adenosine extracellular levels [46,47,48,49,50,51].

#### 2.1.3. Serotonin Receptors

Serotonin (5-HT), an endogenous monoamine neurotransmitter, acts on fourteen different receptors classified into seven different classes (5-HT_1_ to 5-HT_7_) [52]. In particular, the activation of 5-HT_1A_ receptors causes hyperpolarization via Gi/o-protein-coupled potassium channels [52]. CBD is a low-affinity agonist of the human 5-HT_1A_ receptors [53]. However, it has been proposed that the 5-HT_1A_-mediated effects of CBD are more attributable to allosteric interactions with the receptor binding site and/or interference with certain intracellular pathways [54].

#### 2.1.4. Dopamine Receptors

Dopamine, other monoamine neurotransmitter, can act on two types of specific G protein-coupled receptors: D1-like and D2-like dopamine receptors, which activation can elicit different intracellular responses [55]. In this regard, cannabinoids seem to modulate dopaminergic signaling [46,56,57,58,59], and specifically, CBD has shown to have a partial agonistic action on dopamine D2-like receptors [60,61], action that may block the D2 receptors when they are overreactive, or activate them when they are underactive [61].

#### 2.1.5. G-Protein-Coupled Receptor 55 (GPR55)

GPR55 is a seven-transmembrane orphan G-protein-coupled receptor that can be activated by endocannabinoids and by lipid transmitters [62,63]. CBD acts as a suppressor of GPR55 activation, which facilitates an increase in neurotransmitter release [62,64], and has been associated with CBD’s anti-inflammatory effects [65].

#### 2.1.6. Peroxisome Proliferator-Activated Receptors (PPARs)

These receptors belong to the type II nuclear hormone receptor superfamily and three isoforms have been identified: PPARα, PPARβ/ɗ, and PPARү [66]. Specifically, CBD is an agonist of PPARү, and this agonist action is able to induce neuroprotective and anti-inflammatory effects [17,18,67].

#### 2.1.7. GABA Receptors

GABA (main inhibitory neurotransmitter) binds to GABA_A_ receptors promoting a rapid influx of chloride ions and resulting in hyperpolarization and inhibition of the cell [68,69]. Interestingly, GABA_A_ receptors represent a target for CBD, either enhancing inhibitory GABA_A_ receptor activation [70], potentiating GABA-mediated inhibitory currents [71], or enhancing the amplitude of the GABA-evoked currents [72].

#### 2.1.8. Opioid Receptors

The opioid system is integrated by four associated G-protein-coupled receptors [µ (MOR), ɗ (DOR), ҡ (KOR), and the non-opioid, nociceptin/orphanin FQ (NOP)] and diverse opioid neuropeptides [73,74]. CBD behaves as a negative allosteric modulator of MOR and DOR [75] and decreases MOR gene expression [76].

#### 2.1.9. Transient Receptor Potential Vanilloid (TRPV) Channels

TRPV channels are direct regulators of intracellular calcium [77]. TRPV1 is a non-selective cation channel [78] which can be activated by CBD [79,80,81]. This agonist action on TRPV1 channels [43], increases entry of extracellular calcium ions through the channel [82], activating the enzyme calcineurin and dephosphorylating TRPV1 channel, to finally desensitize it [83]. Moreover, CBD can also activate TRPV2 channels [42], which under basal conditions, are located in the endoplasmic reticulum, and once activated, they translocate to the plasma membrane [84]. More recently, a study focused on the role of TRPV2 channels in human brain endothelial cells, pointed out that CBD could be considered as a pharmacological tool to regulate the tightness of the blood brain barrier [85].

#### 2.1.10. Other Ion Channels

Some ion channels being proposed as additional CBD targets include: (a) voltage-gated sodium channels, where CBD decreases the sodium current, although the exact mechanism of interaction is still unknown [86,87]; (b) low voltage-activated T-type calcium channels, inhibited by CBD [88]; and (c) potassium channels that generate M-current, where CBD showed recently to enhance neuronal M-current [89].

## 3. Cannabidiol's Actions for Seizures, Epilepsy, and Drug-Resistant Epilepsy: Preclinical evidence

### 3.1. Mechanisms Underlying Seizures and Epilepsy

Epilepsy is a very complex neurological disease [90]. The lack of a full effective pharmacological treatment despite the availability of a large number of anti-seizure medications (ASMs) and the risk of developing drug-resistant epilepsy are relevant aspects of this disease.

In terms of etiology, epilepsy can have a structural, genetic, infectious, metabolic, immune, or unknown origin [91], although the origin of epileptic seizures is a multifactorial phenomenon.

From a neurochemical perspective, it has been identified that epileptic seizures occur due to an “epileptic discharge”, i.e., the loss in the dynamic balance between inhibitory (mainly by GABA) and excitatory (mainly by glutamate) neurotransmitter systems. Thus, an alteration between these systems promotes the neuronal hyperexcitability and hypersynchronicity distinctive of epilepsy [92,93]. However, the mechanisms involved in the fine modulation of this synaptic dysfunction are multiple and not unique to these two neurotransmitter systems previously mentioned. Changes in the synthesis and release of neurotransmitters, their receptors, molecules involved in their transductional mechanism, as well as alterations in diverse ionic channels have been implicated in epilepsy [93]. Additionally, changes in neuronal connections and circuits (axonal and dendritic plasticity), neuronal cell loss, aberrant neurogenesis, and neuroinflammatory pathways activation, also contribute to the epileptic etiopathogenesis process [94].

### 3.2. Mechanisms Underlying the Anti-Seizure Effects of CBD

As we previously pointed out, CBD is a multitarget molecule and therefore can modulate neuronal excitability. A recent study reported that from CBD's interactions with transmembrane receptors, almost 74% inhibit cell activity, whereas only 26% stimulate it [6]. It is important to highlight that from CBD's interactions and the resulting inhibitory effect on cell activity, CBD’s efficacy as a seizure-controlling drug could be explained.

First, although CBD does not promote direct activation of CB1R, an indirect activation of this receptor results from increasing levels of anandamide due to CBD's actions (FAAH blockage and anandamide cellular uptake inhibition) [42,43]. This effect could suppress neuronal hyperactivity, protecting against seizures [95,96,97]. Moreover, CBD seems to prevent CB1R alterations on limbic brain structures, favoring the control of neuronal activity [98].

CBD can also increase extracellular levels of adenosine by ENT1 blockage [46]. This effect allows activation of A_1_ receptors by adenosine, with anti-seizure effects as a result [99].

On the other hand, although the serotonergic signaling is known to be involved in epilepsy [100] and CBD shows affinity for 5-HT_1A_ receptors [53], the role of these receptors is uncertain in the anti-seizure effects of CBD [101].

In relation to dopamine neurotransmission, since CBD has been proposed as a partial agonist of the D2 dopamine receptor [60,61], it is possible that CBD's anti-seizure effects are due in part to its actions on the dopaminergic system since alterations of this system have been associated as a possible pathophysiological mechanism of epilepsy [102,103,104,105,106].

Another important target is the GPR55 receptor that regulates glutamate release through a calcium-dependent mechanism, modulating neuronal excitability [107]. Therefore, antagonism of GPR55 receptors by CBD can reduce glutamate release and have anti-seizure efficacy [108,109].

Additionally, abnormalities of GABAergic transmission have been associated with epilepsy development [110,111]. CBD has been reported as a positive allosteric modulator of GABA_A_ receptors [71,112], possibly enhancing GABAergic inhibitory transmission and explaining additional CBD’s anti-seizure effects.

With respect to TRPV1 channels, their activation increases neuronal excitability [113] and, in consequence, facilitates seizures [114,115,116,117,118,119]. Although CBD has agonistic actions on TRPV1 channels, it induces a rapid desensitization of these channels [42] that could indirectly decrease the neuronal excitability and explain CBD's anti-seizure effects [120,121].

Finally, the T-type calcium channels and voltage-gated sodium channels are involved in the regulation of cell excitability and therefore they have a role in epilepsy [122,123]. It has been demonstrated that CBD causes the inhibition of these channels [88,124], effects that could mediate the anti-seizure actions of CBD [87,125]. Additionally, CBD has been shown to enhance neuronal M-current, a potassium current whose activation could also underlie CBD's anti-seizure efficacy [89].

### 3.3. CBD and Seizures

In the search of new therapeutic options, CBD has managed to position itself as a promising molecule to treat seizures [126].

In acute seizure models (as chemoconvulsants [3-mercaptopropionic acid, 3-MA; pentylenetetrazol, PTZ; kainic acid, KA; pilocarpine; N-methyl-D-aspartate, NMDA; soman]; electrical stimulation [maximal electroshock, MES; 6 Hz model]; acoustic stimulation, high temperatures or virus infection), CBD has demonstrated its anti-seizure capacity at variable doses (see Table S1).

The anti-seizure effect of CBD has been tested in rodents of different ages, which gives additional information regarding the effect of this cannabinoid in different stages of neurodevelopment. Most of the research performed in adult animals used males, and only one study analyzed the effect of CBD considering the female estrous cycle. The preclinical evidence about the effectivity of CBD in combination with other ASMs is limited. Only few studies have evaluated CBD's effects on combination with classical (phenytoin, clonazepam, midazolam) and other more recent ASMs (tiagabine, lacosamide, oxcarbazepine, gabapentin, levetiracetam, topiramate, pregabalin, lamotrigine). Most results support a synergic effect when CBD and ASMs are combined, but the information is not conclusive. Table S1 briefly summarizes the main findings obtained from these studies that we have consulted and included for this review.

CBD's anti-seizure potential in the MES model is observed at an ED50 of 80–83.5 mg/kg in adult mice and 53.2–68.78 mg/kg in adult rats, avoiding generalized tonic-clonic seizures (GTCS) and increasing the seizure threshold (the electrical current that produces seizures) [127,128,129,130,131]. Additionally, in that model, CBD (100 mg/kg) increases the anti-seizure effect of some ASMs (topiramate, oxcarbazepine, pregabalin) when it is administered in combination with them [130]. Other observed effects with CBD were the attenuation of the anti-seizure efficacy of levetiracetam (100 mg/kg CBD), and lack of effect on the anti-seizure efficacy of lamotrigine or lacosamide [130].

CBD has a protective effect against psychomotor seizures in adult mice at doses of 50–360 mg/kg (ED50 = 144–173 mg/kg) [128,129,130,132,133]. The same effect is observed against hyperthermia-induced seizures in developing and adult mice [109,134,135,136]. For example, Yu et al. [136] found that CBD (3–30 mg/kg) increased seizure latency, reduced seizure severity and the proportion of fourteen postnatal (P) days old (P14) mice that achieved GTCS. A similar effect was observed in P14–16 Scn1a+/− mice (a model of Dravet Syndrome, DS) where CBD (100 mg/kg) managed to increase the temperature threshold for hyperthermia-induced seizures [134].

In the acute audiogenic seizure model of genetic origin, acute administration of CBD (100 mg/kg i.p.,) reduced generalized clonic convulsions, but without entirely abolishing seizures [137].

Chronic CBD treatment (100 mg/kg i.p. bid, 2 weeks) had no significant effects on seizure behavior [137]. Moreover, coadministration of valproic acid (VPA) plus CBD did not alter the therapeutic outcome of VPA as a monotherapy [137].

Another experimental approach extensively used to characterize the effect of CBD on seizure control is the PTZ model in mice and rats [31,108,121,128,129,133,138]. In this model, CBD was able to protect mice (80, 83.5 mg/kg) and rats (88.9 mg/kg) from tonic-clonic seizures in a dose-dependent manner [128,129]. Vilela et al. [121] evaluated CBD pretreatment in different protocols with PTZ. In all experiments, the authors found that CBD (60 mg/kg; i.p.) decreased both seizure frequency and duration. The results by Uttl and colleagues [138] also showed that CBD administration (60 mg/kg) reduced seizure severity and completely blocked the tonic phase in mice. We noticed that actually there is only one study in which female rats were tested and CBD (50 mg/kg) was administered during the proestrus-estrus transition phase [31]. In this study, CBD reduces the duration and severity of PTZ induced seizures, although the latency, incidence of seizures, and mortality rate remained unchanged [31].

Only a couple of reports have evaluated the anti-seizure effect of CBD in models with a primary glutamatergic mechanism: the NMDA-induced seizure model, where CBD's pre-treatment (60 mg/kg) did not protect P12 male rats from seizures [138]; and the intrahippocampal administration of CBD has been reported to be effective to inhibit KA-induced seizures in P20 rats [139].

In pilocarpine-induced behavioral seizures, CBD's pretreatment (1–100 mg/kg) promoted an anticonvulsant effect, increasing the latency and reducing the severity of seizures [140,141].

Finally, in the model of recurrent generalized seizures induced by repetitive application of 3-MA in rats, CBD (200 mg/kg) did not modify the expression of the 3-MA-induced seizures but reduced the incidence of *status epilepticus* (SE). The combination of CBD with phenobarbital reduced the prevalence of SE [142].

All the results previously mentioned obtained from different acute preclinical models support CBD's anti-seizure effect, in both the developing and the adult brain, where acute administration is prevalent and the doses of CBD employed vary between the studies mentioned above. However, there is less information on the effect of combining CBD with ASMs, and it is worth noting that although results suggest mostly a potentiation effect, some studies also show that CBD does not enhance ASMs efficacy or may even promote a pro-seizure effect [130,135,137,142,143]. Further preclinical research is required to evaluate the probable interactions between CBD and currently used ASMs.

### 3.4. CBD, Epilepsy, and Drug-Resistant Epilepsy

The preclinical evidence regarding the effect of CBD in chronic models of epilepsy is still limited (Table S1).

For example, in the pilocarpine epilepsy model, CBD (10 mg/kg i.p.) demonstrated anticonvulsant effects, either by single or multiple administrations, decreasing both SE severity and mortality rate [144], while acute CBD (10 mg/kg i.v.) pretreatment was found to attenuate the maximum seizure severity [129].

In the mouse corneal kindling model, acute administration of CBD (220 mg/kg; i.p.) reduced the frequency of limbic seizures in a dose-dependent manner on kindled mice [128]. Similar results were observed by Patra et al. [129] in the same animal model. CBD also produced partial suppression of both generalized seizures (ED50 = 283 mg/kg) and focal seizures (ED40 = 320 mg/kg) in the amygdala-kindling model [145].

After chronic administration of CBD (50 mg/kg; i.p./daily/28 days/prior to PTZ stimulus) during the PTZ-kindling development, CBD managed to delay the kindling progression although it did not avoid the kindled state [146]. In this same model, another dose of CBD (60 mg/kg; i.p.) was also able to delay the progression of kindling [121]. In another study, daily administration of CBD for 2 weeks (100 mg/kg, i.p., once a day) during the post-kindling phase, did not protect against flurothyl induced seizures, suggesting that CBD could not prevent the pro-epileptogenic process developed in this mouse model of Angelman Syndrome [147]. However, recently, Lazarini-Lopez et al. [98] suggested that CBD could indeed have antiepileptogenic effects, since the chronic administration of CBD (25 mg/kg i.p.; bid, for 10 days) prevented the development of limbic seizures in the audiogenic kindling model, as well as the increase in neuronal activity and the changes in CB1 receptor expression [98].

Finally, CBD's effect in drug-resistant epilepsy has not been thoroughly evaluated (Table S1). For example, in the 6-Hz corneal stimulation model of “psychomotor seizures”, considered as a drug-resistant epilepsy model [148], CBD (164 and 173 mg/kg, i.p.) protected against forelimb seizures and facial clonus [128,129]. On the contrary, in the lamotrigine-resistant amygdala kindling model, CBD (100 and 300 mg/kg, i.p.) did not have any type of protection against epileptic seizures in kindled rats [128].

All the preceding studies performed in chronic models of epilepsy support a partial suppression of seizures by CBD through acute and sometimes chronic protocols of administration. It is necessary to thoroughly research CBD as a disease-modifying agent (by its anti-inflammatory, anti-oxidant, or neuroprotective actions), and its possible coadministration with ASMs. The fasting or fed state of the experimental animal could influence both the brain levels and the anti-seizure effects observed with CBD, a factor that is still undetermined. More preclinical research is required before any conclusions can be made about the therapeutic potential of CBD in drug-resistant epilepsy treatment.

## 4. Other Natural Cannabinoids with Anti-Seizure Potential

Despite the great attention that CBD has received in the search for new therapeutic options for the control of seizures and epilepsy, new interest has emerged in other phytocannabinoids from C. sativa, that have begun to be evaluated as therapeutic options due to their anti-seizure properties observed in preclinical models (see Table S2).

Cannabichromene (CBC), a phytocannabinoid frequently detected in artisanal cannabis oils, and other CBC-related derivatives (5-fluoro-CBC, cannabichromenic acid CBCA, cannabichromevarinic acid CBCVA) have recently shown anti-seizure effects against hyperthermia-induced seizures in Scn1a+/− mouse model of DS [149].

**Figure 2:**
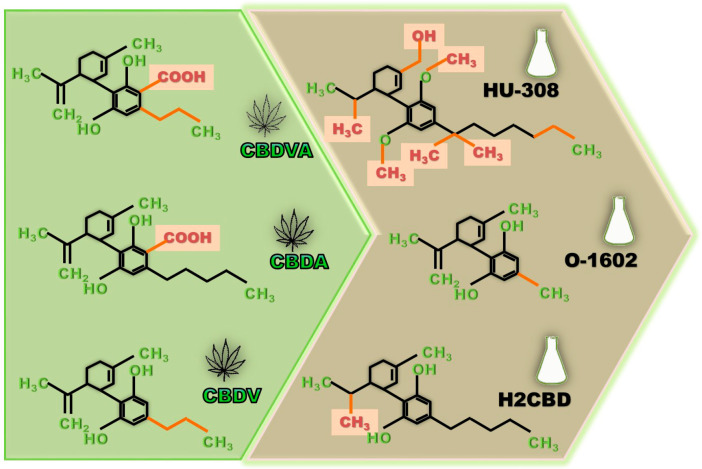
Examples of the chemical structures of some natural cannabinoids (green box) and of synthetic CBD derivatives (brown box) that recently have attracted attention in the search for anti-seizure medications. CBDVA, cannabidivarinic acid; CBDA, cannabidiolic acid; CBDV, cannabidivarin; HU-308, pinene dimethoxy-DMH-CBD derivative; O-1602, a synthetic cannabinoid; H2CBD, 8,9-dihydrocannabidiol.

A similar anti-seizure potential was observed with other phytocannabinoids (also present in artisanal cannabis oils), such as cannabigerolic acid (CBGA), cannabigerovarinic acid (CBGVA), and cannabidivarinic acid (CBDVA) (Figure 2), against seizure activity induced by MES test and against hyperthermia-induced seizures in Scn1a+/− mouse model of DS [150]. It was noticed that CBGA shows superior potency against hyperthermia-induced seizures than CBD, but its anti-seizure effects may depend on the type of preclinical seizure model tested [150] (Table S2). Since CBGA serves as a biosynthetic precursor for CBD [151], it should be taken into account if CBGA's anti-seizure effects are due to CBGA's metabolism or due to the direct effect of CBD.

Moreover, cannabidiolic acid (CBDA) (Figure 2), a phytocannabinoid acid and a precursor of CBD, has recently been shown to also raise the thermogenic seizures threshold in the Scn1a^RX/+^ mouse model of DS [152]. An interesting finding from that study was that CBDA has poor brain penetration when dissolved in vegetable oil (95% canola oil, 5% sunflower oil), but when the drug was dissolved in Tween 80-based vehicle (ethanol-Tween 80-saline; 1:1:18 ratio) its brain concentration increased [152].

These data emphasize the importance of selecting adequate vehicle preparations for cannabinoids. However, because exposure to heat or light decarboxylates CBDA into CBD [153], magnesium ions have been used to stabilize CBDA-enriched hemp extracts (Chylobinoid and Mg-CBDA) [154] (Table S2). Both extracts demonstrated anti-seizure effects at the MES test in rats [154]. Another study using a CBD/CBDA-rich hemp extract oil showed a reduction in the frequency of canine refractory epileptic seizures during three months of treatment [155].

Cannabidivarin (CBDV) (Figure 2), a propyl analog of CBD, has been evaluated as a purified drug or as a cannabis extract rich in CBDV/CBD, due to its anti-seizure properties observed at the preclinical level [151,156,157]. In a model of acute seizures, CBDV (400 mg/kg oral gavage) decreased PTZ-induced seizure severity and increased onset latency, effects that were associated with changes in the expression levels of some epilepsy-related genes [156]. CBDV in vitro, suppressed epileptiform activity in brain slices, while in vivo had anti-seizure effects on several preclinical models [151,157].

Moreover, CBDV suppressed PTZ-induced seizures in P10 rats, while in P20 rats, CBDV suppressed seizures in several models, including the PTZ, MES, and DMCM (methyl-6,7-dimethoxy-4-ethyl-beta-carboline-3-carboxylate) models. Due to the above, it is suggested that CBDV could have therapeutic value as a novel treatment for childhood epilepsy [158] (Table S2).

As a major metabolite of CBD, 7-OH-CBD has also exerted anti-seizure effects in the MES model in mice [159].

Besides CBD, other compounds such as cannabinol (CBN) and linalool (LN, a cannabis's common terpene) have reduced seizure behavior in the Scn1Lab−/− mutant zebrafish, a DS model [160] (Table S2). Still, further research of their anti-seizure potential is necessary.

Other examples of natural cannabinoids are two olive oil hemp extracts (K1, without volatile compounds; K2; extract rich in terpenes) derived from *Cannabis sativa* L. (highly enriched in CBD) that reduced the occurrence of convulsive seizures in the 6-Hz corneal stimulation mouse model, where it is suggested that terpenic components could enhance the anti-seizure activity of cannabinoids contained in the K2 oil hemp extract [161] (Table S2). These findings suggest not only that both cannabinoids and terpenes found in oil extracts could influence the anti-seizure effects observed, but also that oil extracts could have potential therapeutic effects against seizures, epilepsy, or drug-resistant epilepsy.

Some other CBD analogues [162] awaiting pharmacological evaluation for their therapeutic benefits include: cannabinodiol (CBND-C5) and cannabinodivarin (CBND-C3), two aromatic CBD's analogs isolated from Lebanese hashish [163]; and cannabielsoin (CBE), a cannabinoid metabolite identified in plants as a product of photo-oxidation from CBD and CBDA [164].

In relation to clinical trials, there are two recent studies that assessed CBDV's efficacy [165,166]. In one study, add-on CBDV (400 to 800 mg b.i.d./2 w + 800 mg b.i.d./6 w) treatment in adult patients with focal seizures, there was no change in seizure frequency between the CBDV and placebo treatment groups, after 8 weeks of treatment [165]. The other study included five girls with Rett Syndrome, where CBDV (oral solution, 50 mg/mL; 10 mg/kg/day) induced a trend toward reduction in mean monthly seizure frequency [166].

Further pharmacological characterization of the biological effects at the preclinical level of the phytocannabinoids reviewed here as well as of the cannabis extracts is necessary, in order to assess whether or not these compounds could be considered in the future as new therapeutic options to improve seizures, epilepsy, or drug-resistant epilepsy outcomes, besides the already known ASMs.

## 5. Synthetic CBD Derivatives

Other pure molecules, with therapeutic potential, are the so-called synthetic CBD derivatives, which have major potency, efficacy, or pharmacokinetic profile (Figure 2) [162]. Structural modifications of the CBD's molecule include: the alkyl side-chain, the propylcyclohexane, substitution of the phenolic hydroxyl groups, and quinone based synthetic analogs [162].

The pharmacological activity of synthetic CBD derivatives has recently been started to be investigated in a few acute seizure models.

For example, the 1,1-dimethylheptyl (DMH)-CBD derivatives as the pinene dimethoxy-DMH-CBD derivative (HU-308), or the O-1602 (another synthetic cannabinoid), have not shown anti-seizure effects against clonic seizures in the DMCM model (Figure 2) [167].

On the other hand, the 8,9-dihydrocannabidiol (H2CBD), a fully synthetic CBD's analogue, exhibited a dose-dependent anti-seizure action in acute PTZ-induced generalized seizures in rats, with a protective effect comparable to CBD (Figure 2) [168]. Because H2CBD is fully synthetic, it has been suggested that this molecule could not be considered a controlled substance and therefore could circumvent legal issues surrounding cannabis-based therapies [168].

There is only one clinical study that for the first time reported a reduction of seizure frequency in drug-resistant epileptic patients due to treatment with a pharmaceutical grade, synthetically derived CBD [169].

Further preclinical research (in acute and chronic animal models) regarding these synthetic molecules for more precise characterization is required in order to establish whether they have or not anti-seizure or antiepileptogenic effectivity, a better or comparable effectiveness to natural derived CBD, and how they could have or not therapeutic potential in drug-resistant epilepsy.

## 6. Future Perspectives

### 6.1. Mitochondrial Dysfunction, Epilepsy, and CBD

Mitochondria has critical cellular functions as the generation of energy (ATP), calcium homeostasis, and control of cell death. Moreover, it is the primary site of reactive oxygen species (ROS) production [170].

In general, mitochondrial dysfunction includes depletion of ATP, the blockade of enzymatic activity in the electron transport chain, generation of ROS, decrease of mitochondrial DNA (mtDNA), calcium imbalance mechanisms and alteration of apoptotic pathways [171]. All these events triggered by mitochondrial dysfunction increase neuronal excitability and promote cellular damage [172]. Therefore, mitochondrial dysfunction has been proposed as an active contributor to the onset and progression of epilepsy [172,173,174,175,176,177,178,179,180,181,182].

In this regard, CBD seems to have the capacity to reduce the intracellular calcium concentration when it is abnormally elevated as in excitotoxic circumstances, via direct interaction with the mitochondrial Na^+^/Ca^2+^ exchanger (NCX) [183]. The effect of CBD over the intracellular calcium concentration could prove to have anti-seizure properties by restoring the calcium balance and therefore decreasing neuronal excitability, especially relevant during mitochondrial dysfunction associated with epilepsy [171,179]. CBD also has a direct interaction with the outer-mitochondrial membrane protein, the voltage-dependent anion channel 1 (VDAC1), interaction that in this case increases intracellular calcium concentrations in BV-2 microglial cells [184]. The above suggests that CBD's mitochondrial regulation of the intracellular calcium concentration could be a cell type-dependent effect [185].

On the other hand, it has been demonstrated that not only CB1 receptors are present in brain outer mitochondrial membranes [186,187], but also it has been corroborated their participation in the inhibition of brain mitochondrial respiration [188]. Specifically, CBD has been reported as a full inhibitor of mitochondrial respiration through inhibition of electron transport chain complexes I, II/III, and IV [188,189]. However, it has been suggested a biphasic effect of CBD on mitochondrial respiration [185], where low concentrations could activate mitochondrial respiration [190] and high concentrations could inhibit it [188,189,191]. This is quite relevant since the modulation of mitochondrial respiration could lead to either neuroprotective or neurotoxic effects, affecting both brain mitochondrial metabolism and neuronal integrity, which may contribute to the onset and progression of epilepsy.

Mitophagy, a selective autophagy of mitochondria, is an important mitochondrial mechanism that eliminates dysfunctional or damaged mitochondria to maintain mitochondrial homeostasis and cell survival [192]. Interestingly, defects in mitophagy due to mitochondrial dysfunction have begun to be studied as a pathophysiological mechanism of epilepsy [193,194,195,196].

Recently, it has been reported that CBD activates the PINK1/Parkin pathway in a dose-dependent manner [197]. This pathway initiates not only the mitophagy process, but also the production of mitochondrial derived vesicles (MDV; to transport damaged proteins and lipids to lysosomes) [198,199]. How these CBD's actions over the PINK1/Parkin pathway can have therapeutic value during mitochondrial dysfunction and consequently in epilepsy progression, are questions that remain to be clarified.

Further research is needed that focus on the effects of CBD over specific cell types (e.g., neurons, microglia, astrocytes), as well as an evaluation of the concentrations and doses required for the desired therapeutic effects of CBD, with respect to mitochondrial dysfunction.

If further research proves that CBD can indeed modulate mitochondrial dysfunction, then this would be a different mechanism via which CBD could be a promising tool to treat epilepsy.

### 6.2. Mammalian Target of Rapamycin, Epilepsy, and CBD

The mammalian target of rapamycin (mTOR) can affect neuronal signaling and excitability, neurotransmitter receptor expression, and synaptic plasticity [200]. This signaling pathway has been proposed as a pathophysiological mechanism in epilepsy [200], since hyperactivated signaling has been reported in both preclinical models as well as in human tissue resected from epileptic patients [201,202,203,204,205,206,207,208,209].

New findings have found that the genetic deletion of PI3Kγ abolishes not only CBD's anti-seizure effects but also CBD's neuroprotection against pilocarpine-induced seizures in mice [141]. The inhibition of mTOR, which is an important component of the PI3K pathway, also blocked CBD's anti-seizure effects [141]. These results suggest that some CBD's actions could be mediated by the PI3K/mTOR pathway [141]. Further studies are required to establish the therapeutic implications of this new mechanism of CBD, in both epilepsy and drug-resistant epilepsy.

## 7. Cannabidiol: Clinical Evidence

Nowadays, the only brand version of CBD approved by the US Food and Drug Administration (FDA) and European Medicines Agency (EMA), and indicated for the treatment of severe epilepsy syndromes (Dravet Syndrome, DS; Lennox-Gastaut Syndrome, LGS) and for tuberous sclerosis complex (TSC) [32,34,210,211], is the highly purified CBD formulation Epidiolex^®^/Epidyolex^®^ (oral solution in sesame oil) [212].

Since then, diverse clinical trials have been conducted to evaluate the safety, tolerability, and efficacy of CBD in humans, mainly in patients with severe epilepsy syndromes and TSC, diseases associated with drug resistant seizures in children and young adults [32,34,211,213,214,215,216,217,218,219].

In these trials, CBD has demonstrated an improvement in the patient’s overall condition as adjunctive treatment, by observing a decrease in the seizure frequency. Patel et al. [220] recently reported that CBD does not lose its efficacy in controlling seizures over a treatment period of up to 60 months, in patients with drug-resistant epilepsy of various underlying etiologies (children, young adults, adults). Moreover, other study showed that CBD's efficacy lasts over two years of treatment, in adults and children with drug-resistant epilepsy [221].

On the other hand, CBD treatment has been well tolerated, with adverse events mostly mild or moderate in severity, across different populations including children, adolescents, and younger adults, during the treatment of drug resistant seizures associated with these severe forms of epilepsy. A recent study reported the first systematic review and meta-analysis of the adverse effects of CBD across a range of medical indications [222]. These clinical trials data were mainly obtained from childhood epilepsy syndromes, and suggest that CBD is well tolerated and has few adverse effects, despite the high doses used of CBD [222]. Serious adverse events related to abnormal liver function tests (increased liver transaminases, a sign of hepatotoxicity) or pneumonia were reported. Moreover, other adverse events reported were diarrhea, reduced appetite, and somnolence [222]. It has been estimated that the time to onset of CBD treatment effect (seizure reduction and adverse events), may occur within the first week of treatment and most adverse events resolved within 14-week period (e.g., in patients with LGS) [223].

More information can be consulted through the US National Institutes of Health Clinical Trials Registry (http://www.clinicaltrials.gov, accessed on; searching Epilepsy + CBD), or it can consult some recent reviews of this topic [224,225,226,227,228].

## 8. Conclusions

The cannabidiol compound has gained popularity in the search for new therapeutic options to treat seizures, epilepsy, or drug-resistant epilepsy. However, CBD's medical use has been restricted since it is still a controlled substance in many countries worldwide.

Evidence obtained from preclinical studies highly support CBD’s anti-seizure effect, which involves neuronal excitability modulation throughout different mechanisms.

Our review summarizes new current data that should be taken into account in future research to investigate the anti-seizure potential of CBD, and lately, other natural and synthetic cannabinoids, from mechanisms of action and pharmacological profiles to innovative formulations (e.g., the molecule's protection from heat or light by using magnesium ions).

Of relevance, due to the fact that CBD is a poor water-soluble molecule, its dissolution in different vehicle preparations, oil-based, surfactants or non-ionic surfactants, may promote differences in the final concentration of CBD in both plasma and brain, highlighting the importance of vehicle selection. In addition, other factors that may affect CBD’s final brain levels include molecule's purity, used dosage, route of administration, fed state, and sex. Potential differences in CBD efficacy could be influenced by the animal species and seizure or epilepsy model chosen. CBD's drug interactions with ASMs should be the main goal for further research in order to avoid toxic or counterproductive effects after chronic treatment with CBD.

Regarding the clinical application of CBD, both its efficacy and optimal doses for the treatment of other epileptic conditions such as temporal lobe epilepsy (a known type of drug-resistant epilepsy) or status epilepticus, in populations of adult patients, remain to be investigated. Many questions still remain unsolved and further research is necessary before considering CBD and other cannabinoids as new therapeutic choices (e.g., monotherapy, adjunctive therapy) with potential to treat not only seizures or epilepsy, but hopefully drug-resistant epilepsy as well in adult patients.

## Data Availability

Not applicable.

## Supplementary Materials

The following supporting information can be downloaded at: https://www.mdpi.com/article/10.3390/ijms232416181/s1.
