# Supplementary material for: Advances and Challenges of Cannabidiol as an Anti-Seizure Strategy: Preclinical Evidence
Source: Int J Mol Sci. 2022 Dec 19;23(24):16181. doi: 10.3390/ijms232416181 (PMC9783044; doi:10.3390/ijms232416181)
Supplement: Supplementary file 1 [file ijms-23-16181-s001.zip › Supplementary Materials-Table-S1-S2-ijms-2031481-final.pdf]

Supplementary Materials.

Advances and challenges of cannabidiol as an anti-seizure strategy: preclinical evidence.

Cecilia Zavala-Tecuapetla[1,*], Hiram Luna-Munguia[2#], María-Leonor López-Meraz[3#] and Manola Cuellar-Herrera[4#]

[1] Laboratory of Physiology of Reticular Formation, National Institute of Neurology and Neurosurgery. Insurgentes Sur 3877, La Fama, 14269, Mexico City, Mexico; cztecua@yahoo.com.mx
[2] Departamento de Neurobiologia Conductual y Cognitiva, Instituto de Neurobiologia, Universidad Nacional Autonoma de Mexico. Campus UNAM-Juriquilla, 76230, Queretaro, Mexico
[3] Instituto de Investigaciones Cerebrales, Universidad Veracruzana. Luis Castelazo Ayala s/n, Col. Industrial Á nimas, 91190, Xalapa, Veracruz, México
[4] Epilepsy Clinic, Hospital General de México Dr. Eduardo Liceaga; Dr. Balmis 148, Doctores, 06720, Mexico City, Mexico

Table S1. Studies that tested the anti-seizure effects of cannabidiol (CBD) on Acute Seizure and Epilepsy preclinical models. This table includes 26 studies concerning acute seizures or epilepsy animal models, ordered by year, from 2001 until 2022.

Table S2. Natural cannabinoids tested in preclinical seizure models. This table includes 11 studies from 2012 to 2022, ordering based on the compound evaluated, concerning natural cannabinoids and acute seizures animal models.

It should be noted that the information in these tables not only includes the experimental model evaluated and the main findings, but also the dose, route of administration, type of administration (acute or chronic), administration times, and also it is indicated if there is co-administration with other ASMs, the type of compound used, and most important, the vehicle selection. All these data that are shown in a simplified manner will give the reader a general idea of what has been researched up to now and what still needs to be done in this field.

References are included at the list of the main text.

Table S1. Studies that tested the anti-seizure effects of cannabidiol (CBD) on Acute Seizure and Epilepsy preclinical models.

Model	CBD (alone/combination)	Effect	Reference
**MES**<br>50 mA current/ 0.2 s / pulse train of 60 Hz/ corneal electrodes<br>CF-1 male mice	CBD (NIH) i.p.<br>veh: etanol/Emulphor-620/0.9% saline (1:1:18)<br>2 h prior to MES	Anticonvulsant effect ED_{50} = 80 mg/kg	**[127]**
In vitro<br>**Mg^{2+}-Free**<br>**4-AP (100 µg)**<br>Transverse hippocampal brain slices (450 µm)<br>P21 Wistar Kyoto rats<br>***In vivo***	CBD (GW Pharmaceuticals)<br><br>*In vitro*<br>veh: DMSO<br>0.01, 0.1, 1, 10, 100 µM<br><br>*In vivo*	🡻Recurrent SE-like LFP events burst amplitude and burst duration in the CA1, CA3 and DG (0.1-100 µM)<br><br>Suppressed 4-AP-induced epileptiform LFP burst amplitude (100 uM)	**[108]**

**PTZ induced seizures**<br>80 mg/kg<br>Adult male Wistar Kyoto rats	veh: ethanol/Cremophor/0.9% saline (1:1:18)<br>1, 10, 100 mg/kg, i.p.<br>60 min prior to seizure induction	↓Latency to GTCS (1 mg/kg), ↓seizure severity (100 mg/kg), ↓proportion of animals with GTCS, ↓mortality	
**Pilocarpine induced seizures**<br>380 mg/kg; i.p. 15 min after CBD<br>**Penicillin model**<br>525 IU penicillin (right lateral ventricle); 1 h after CBD<br>Adult male Wistar Kyoto rats	CBD (GW Pharmaceuticals)<br>veh: ethanol/Cremophor/0.9% saline (1:1:18)<br>1, 10, 100 mg/kg, i.p.	↓%animals with seizures; ↓seizure frequency (1, 10, 100 mg/kg) with pilocarpine<br><br>No changes on seizure severity or occurrence, ↓mortality (10, 100 mg/kg) with penicillin	**[140]**
**PTZ kindling**<br>35 mg/kg i.p./daily/28 days<br>Adult male Sprague-Dawley rats	CBD (Sigma)<br>veh: not mentioned<br>10, 20, 50 mg/kg, i.p.<br>1 h prior to seizure induction	20 and 50 mg/kg<br>↓ severity of PTZ-induced chronic seizures<br><br>delay the progression of PTZ kindling not avoided kindled state	**[146]**
**Hyperthermia induced seizures**<br>Body temperature:<br>36.5 °C/1h; ↑ 0.5 °C/every 2min/ up to 38 °C-30min<br>P21-P28 Scn1a mutant mice	CBD (GW-Pharmaceuticals)<br>veh: ethanol/Cremophor/0.9% saline (1:1:18)<br>10, 20, 50, 100, 200 mg/kg i.p.<br>1h prior to seizure induction	100 and 200 mg/kg<br>Reduced duration and severity of seizures	**[109]**
**Pilocarpine induced SE**<br>1 µl; 2.4 mg/mL i.h.<br>Adult Wistar male rats	CBD (STI Pharmaceuticals)<br>veh: 98% saline/2% Tween 80<br>10 mg/kg i.p.<br>1 h prior to SE induction/ every 12 h after SE induction	↓number of rats with SE<br>↑ latency to SE<br>↓ SE severity and mortality rate	**[144]**
**PTZ induced seizures**<br>60 mg/kg i.p./s.c.<br>10mg/ml i.v. (infusion rate 0.2ml/min)<br>Adult Male Swiss mice	CBD (THC-Pharma)<br>veh: 98% saline/2% Tween 80<br>30, 60, 90 mg/kg<br>30 min prior to PTZ	Pretreatment (60 mg/kg) attenuated seizures	**[121]**
**MES**<br>60 Hz-alternating current/2 s/corneal electrodes/current intensity: 50 mA-mice; 150 mA-rats<br>**6 Hz psychomotor seizures**<br>6 Hz/3 s/corneal electrodes/44 mA/mice<br>**PTZ induced seizures**<br>85 mg/kg, s.c., mice	Synthetic CBD (NIDA Drug Supply Program)<br>veh: ethanol/Cremophore® EL/PBS (1:1:18)<br>50-220 mg/kg i.p.<br>2 h prior to seizure induction	Dose-dependent protection on mice, in both models:<br>6 Hz seizure (ED_{50} = 164 mg/Kg) and MES (DE_{50} = 83.5 mg/kg)<br><br>Anticonvulsant effect in the PTZ mouse model (ED_{50} value of 159 mg/kg)	**[128]**

**Corneal kindling**<br>3 s, 3 mA, 60 Hz corneal stimulation<br>**Lamotrigine-resistant amygdala kindling**<br>200 µA stimulus/daily; Lamotrigine (5 mg/kg, i.p.)/ daily prior to stimulation<br><br>CF-1 male mice<br>Male Sprague–Dawley rats		Dose-dependent protection in the rat MES model (ED_{50} = 88.9 mg/kg)<br><br>Dose-dependent seizure protection in the mouse corneal kindling model (ED_{50} = 119 mg/kg)<br><br>100, 300 mg/kg did not produce seizure protection on lamotrigine-resistant amygdala kindled rats	
**KA induced seizures**<br>5 µM i.h. / 7-8 mg/kg i.p.<br>P20 male Sprague-Dawley rats	CBD (Millipore Sigma-Aldrich) predissolved in methanol<br>3.18 µM i.h.<br>10 mg/kg i.p.<br><br>- Coadministration or 30 min after KA i.p.<br>- Coadministration with intrahippocampal KA	Intrahippocampal coadministration prevented the development of stage 4-5 seizures<br><br>CBD (8 mg/kg) after KA, quickly calmed seizures<br><br>Intrahippocampal coadministration was most effective in inhibiting KA-induced seizures.	**[139]**
**MES**<br>Corneal stimulation<br>Stimulus: 50 mA, 60 Hz, 0.2 s (mice); 150 mA, 60 Hz, 0.2 s (rats)<br>**6 Hz psychomotor seizures**<br>Corneal stimulation<br>two currents: 32 mA and 44 mA; 3 s duration; mice<br>**PTZ induced seizures**<br>85 mg/kg s.c.; mice<br>**Corneal kindling**<br>Twice daily corneal stimulation<br>**Pilocarpine induced SE**<br>380 mg/kg i.p.; rats<br><br>Male Sprague-Dawley rats<br>Male Wistar rats<br>Male CF-1 mice	CBD (GW Pharmaceuticals)<br>veh: Cremaphor/ethanol/water (1:1:18)<br>Various doses of CBD i.p.<br><br>CBD (10 mg/kg, i.v.)<br>veh: kolliphor HS/glycerol anh/EDTA/ascorbic ac/monothioglycerol/water<br>1 h prior to pilocarpine<br><br>CBD (200 mg/kg, oral/ 8w)<br>veh: 3.5% Kolliphor® HS	Protective effect ED_{50}:<br>MES mice = 80 mg/kg<br>MES rat = 53.2 mg/kg<br>6Hz 32mA mouse = 144 mg/kg<br>6Hz, 44 mA, mouse = 173 mg/kg<br>PTZ = 120 mg/kg<br>Corneal kindling = 144 mg/kg<br><br>Attenuated maximum seizure severity in pilocarpine-induced SE<br><br>On post-SE animals, disease severity was reduced by chronic oral CBD	**[129]**
**TMEV induced acute seizures**<br>Mice infected intracortically with 2 x 10^5 PFU-DA TMEV<br>Acute seizures by briefly agitating the mice by shaking their cages<br>**6 Hz psychomotor seizures**<br>Corneal stimulation<br>6 Hz, 0.2 ms rectangular pulse width, 3	CBD (NIDA)<br>veh: 100% Ethanol/ Kolliphor®/0.9% saline (1:1:18)<br><br>180 mg/kg i.p./ bid/10 days<br><br>50 mg/kg, i.p./ 0.5, 1, 2, 4 h	180 mg/kg (360 mg/kg/day)<br>↓ TMEV-induced acute seizure frequency and severity<br><br>150 mg/kg<br>↓ TMEV-induced acute seizures at 2 and 4 h post-treatment	**[132]**

s duration, 32 mA current intensity<br><br>Adult male C57BL/6J mice	prior to corneal stimulation	Anti-seizure effect in the 6 Hz 32 mA model was observed at 2 h post-treatment	
**Hyperthermia induced seizures**<br>In kindled AS model mice<br>Body temperature increased by 0.3°C/min until behavioral seizures scored 3 or core body reached 42.5°C.<br>**Acoustically induced seizures**<br>(Angelman sindrome mouse model)<br>Acoustic stimuli: 1 min by alarm sirens (~125 dB).<br>129 mice, B6 mice<br>AS model mice (Ube3am–/p+)/ wild-type mice	Synthetic CBD (99.2% ± 0.18% purity; RTI International)<br>veh: Ethanol/cremophor/0.9% saline (2:1:17)<br><br>100 mg/kg; i.p.<br>1 h prior to hyperthermia induced seizures<br><br>10, 20, 50, 100 mg/kg; i.p.<br>Acoustically induced seizures	100 mg/kg ↓frequency and severity of seizures triggered by acoustic stimuli<br><br>100 mg/kg ↓hyperthermia-induced generalized seizure duration and severity in the kindled AS model mice	**[147]**
**MES**<br>Transcorneal electrodes<br>sinusoidal alternating current (50 Hz; maximum output voltage 500 V, stimulus duration 0.2 s)<br>**6 Hz psychomotor seizures**<br>Transcorneal electrodes<br>square-wave alternating current stimuli (pulse width 0.2 ms, duration 3 s, frequency 6 Hz)<br><br>Adult male albino Swiss mice	CBD (THC Pharm GmbH)<br>veh: 1% Tween 80<br>25, 50, 100 mg/kg i.p.<br>60 min prior test<br><br>ASMs i.p. prior test:<br>tiagabine - 15 min<br>lacosamide/oxcarbazepine - 30 min<br>gabapentin/ levetiracetam/lamotrigine/topiramate - 60 min<br>pregabalin-120 min	50 and 100 mg/kg, ↑ threshold for 6 Hz- induced psychomotor seizures and MES<br><br>All ASMs had protective effect against 6 Hz-induced psychomotor seizures and MES<br><br>6 Hz- induced psychomotor seizures:<br>50 and 100 mg/kg, ↑ activity of tiagabine and gabapentin<br>100 mg/kg ↓ activity of levetiracetam<br><br>MES:<br>100 mg/kg, ↑ activity of topiramate, oxcarbazepine and pregabalin	**[130]**
**Pilocarpine induced seizures**<br>Bilateral i.h. microinjection (30 μg/0.25 μl/site)<br>Adult male C57Bl/6 wild-type and PI3Kγ-/- mice	CBD (THC-Pharma)<br>veh: 2 % Tween 80<br>30, 60, 90 mg/kg; i.p.<br>60 min prior to pilocarpine	30, 60, 90 mg/kg<br>↑ latency and ↓ severity of pilocarpine-induced seizures, ↓ number of animals with seizures	**[141]**
**Hyperthermia induced seizures**<br>Body temperature increased 0.5 °C/2 min<br>(until first clonic convulsion-loss of posture or until 42.5 °C was reached)<br>P14-16 male and female Scn1a+/− mice	CBD (THC-Pharma)<br>veh: etanol/Tween-80/saline (1:1:18)<br>12, 25, 100 mg/kg, i.p.<br>60 min prior to hyperthermia induced seizures	100 mg/kg ↑ temperature threshold for hyperthermia-induced seizures<br><br>Sub-chronic CBD oral treatment did not affect spontaneous seizure frequency or survival of mice<br><br>Co-administration of Δ9-THC (0.1	**[134]**

	Chow: 3500 mg/kg, 7000 mg/kg<br>daily oral dose (sub-chronic) from P19 to P21 or P30	mg/kg) and CBD (12 mg/kg) ↑ temperature threshold for hyperthermia-induced seizures	
**MES**<br>Single corneal electroshock (0.1 sec)<br>“up and down” method<br>TRPV1 knockout and wild type mice	CBD (GW Pharmaceuticals)<br>veh: etanol/KolliphorEL/saline (1:1:18)<br>10, 25, 50, 100mg/kg, i.p.<br>60 min prior to MES test	50, 100 mg/kg ↑ seizure threshold in wild type animals, and only partially in TRPV1 knockout mice	**[131]**
**LPS induced acute febrile seizure**<br>Single dose, 10 mg/kg, i.p./2 h prior to febrile seizures induction<br>Housing 15 min in a pre-heated 42ºC incubator<br>P14 C57 BL/6 mice	CBD (Shanghai Aladdin Bio-Chem Technology)<br>veh: corn oil<br>3, 10 or 30 mg/kg, i.p.<br>1 h prior to hyperthermic stimulation	3, 10, 30 mg/kg<br>↓ seizure severity, ↑ seizure latency<br>↓ proportion of mice with GTCS<br>animals recovered quickly from seizures	**[136]**
**3-MA induced recurrent generalized seizures**<br>30-37.5 mg/kg i.p./bid/5 days<br>Adult male Wistar rats	CBD (HempMeds)<br>50 or 200 mg/kg, oral<br>2 h prior to 3-MA stimulation<br><br>CBD+ phenobarbital (PB, 15 mg/kg i.p.)<br><br>CBD+ phenytoin (PHT, 75 mg/kg, i.p)	CBD<br>↓ SE prevalence<br><br>CBD+PB<br>↑ seizure latency, ↓ major seizures<br>↓ SE prevalence (drugs applied during seizure induction)<br><br>CBD+PHT<br>↓ major seizures prevalence<br>↑ SE prevalence (drugs applied after seizure induction)	**[142]**
**Soman (pinacolyl methylphosphonofluoridate)-induced seizures**<br>80 µg/kg; s.c.<br>Female Es1-/- mice	CBD (Cayman)<br>veh: 5% etanol/5% Kolliphor EL/<br>90% saline<br>20, 80 or 150 mg/kg; i.p.<br>60 min prior to Soman<br><br>Midazolam (5 mg/kg; i.p.)<br>30 min after seizure onset	150 mg/kg<br>↑ survival rate in soman-exposed mice<br><br>150 mg/kg + midazolam<br>↓ seizure severity in soman-exposed mice	**[143]**
**Hyperthermia induced seizures**<br>Body temperature increased 0.5 °C/ each 2-min (until GTCS or 41 °C was reached)<br>P21-P28 male and female Scn1a+/- mouse	CBD (Cayman Chemical)<br>veh: 1% etanol/1% cremophore/18% saline<br>100 mg/kg, i.p<br>1 h prior to seizure induction<br><br>Clonazepam (CLZ)	100 mg/kg<br>↓ seizure duration<br><br>100 mg/kg + CLZ<br>↑ threshold temperature and<br>↓ duration of thermally induced seizures	**[135]**

	0.0625 mg/kg, i.p.<br>0.5 h prior to seizure induction		
**GASH/Sal audiogenic seizures**<br>Cylindrical acrylic arena (height: 50 cm, diameter: 37 cm); acclimatation/1min continuous white noise (0 to 18 kHz), intensity of 115 to 120 dB until initiation of wild-running phase or until 20 s had elapsed<br>Adult male GASH/Sal hamsters (4 months)	CBD (RiverForce Partners Inc.)<br>veh: Cremophor RH-40/ethanol/saline (1:2:17)<br><br>Acute administration:<br>100 mg/kg i.p.<br>45 min prior to seizure induction<br><br>Chronic administration:<br>7 or 14 days<br>100 mg/kg i.p.<br>daily/ bid /2 w<br><br>Valproic acid (VPA) 300 mg/kg i.p.	Acute treatment:<br>100 mg/kg blocked generalized clonic convulsions<br>100 mg/kg+VPA eliminated the seizure behaviors<br>🡻 duration of sound induced seizures<br><br>Chronic treatment:<br>100 mg/kg or 100 mg/kg+VPA had no effects on seizure behavior	**[137]**
**Amygdala kindling**<br>Stimulation: 1-s train, 60-Hz biphasic square-wave pulses, 1-ms duration, 400 μA current intensity.<br>Daily stimulation (5 days/w) until 10 Stage 5<br>Adult male Sprague Dawley rats	CBD (MedReleaf)<br>Veh: 95% etanol/Cremophor EL (1:1)<br>40, 80, 160, 320 mg/kg i.p.<br>2h prior to seizure stimulation<br><br>CBD + THC<br>15:1 combination<br>40 + 2.66/ 80 + 5.33/ 160 + 10.66 mg/kg i.p.<br>THC 1h prior to seizure stimulation	40, 80, 320 mg/kg had anti-seizure effects against generalized seizures on kindled animals<br>ED50=280 mg/kg<br><br>160, 320 mg/kg had anti-seizure effects against focal seizures on kindled animals<br>ED40 = 320 mg/kg<br><br>Addition of small amounts of THC greatly improves the effectiveness of CBD	**[145]**
**Audiogenic kindling (AuK)**<br>Rat placed into an acoustically isolated chamber; acoustic stimulation (110-120 dB; 5-20 kHz)/1 min or until development of tonic seizure<br>Two acoustic stimulation/day/10 days<br>Adult male WARs rats; Wistar rats	CBD<br>(99.6% purity; BSPG-Pharm-Sandwich)<br>veh: 98% saline/2% Tween 80<br>25 mg/kg; i.p<br>chronic treatment: bid/10 days<br>1 h prior to acoustic stimulation	Chronic treatment:<br>attenuated brainstem seizures, suppressed limbic seizures during the AuK, suggesting antiepileptogenic effects	**[98]**
**PTZ induced seizures**<br>100 mg/kg; s.c.	CBD (THC-Pharm GmbH)<br>veh: 2% Tween	10 or 60 mg/kg did not protect against NMDA induced seizures	**[138]**

**NMDA induced seizures**<br>60 mg/kg; i.p.<br>P12 male Wistar albino rats	20/phosphate buffer (0.001M, pH 7.4)<br>10 or 60 mg/kg; i.p.	60 mg/kg ↓ seizure severity, ↑ latency to GTCS, blocked tonic phase of PTZ induced seizures	
**6 Hz seizures**<br>corneal stimulation (6 Hz, 0.2 ms pulse width, 3 s).<br>16 or 32 mA<br>**PTZ induced seizures**<br>100 mg/kg; s.c.<br>Male RL/+ mutants mice<br>Wild-type mice	CBD (Cayman Chemical)<br>veh: 100% etanol/cremophore/0.9% saline (1:1:18)<br>200-360 mg/kg, i. p.<br>2 h prior to seizure induction	6 Hz seizures:<br>320, 360 mg/kg (16 mA) ↑ resistance to 6 Hz seizures (44%, 83%, respectively)<br><br>360 mg/kg (32 mA) protected 42% against 6 Hz seizures<br><br>PTZ:<br>200-360 mg/kg, ↑ seizure latency, did not avoid GTCS generation	**[133]**
**PTZ induced seizures**<br>60 mg/kg i.p.<br>Adult female Sprague–Dawley rats<br>Proestrus-estrus phase	CBD (donation)<br>veh: 10% DMSO/90% PBS in etanol/KolliphorEL/NaCl 0.9% (1:1:18)<br>50 mg/kg, i.p.<br>1 h prior to seizure induction	50 mg/kg ↓duration and severity of seizures	**[31]**

CBD, cannabidiol; ASMs, anti-seizure medications; ED_{50} = median effective dose; MES, maximum electroshock seizures; PTZ, pentylentetrazole; KA, kainic acid; NMDA, N-metil-D-aspartate; SE, *status epilepticus*; GTCS, generalized tonic-clonic seizures; 3-MA, 3-mercaptopropionic acid; TMEV, Theiler's murine encephalomyelitis virus; 4-AP, 4-aminopyridine; LFP, Local Field Potential; Δ9-THC, Δ9-tetrahydrocannabinol; AS, Angelman syndrome; LPS, Lipopolysaccharide; veh=vehicle; i.p. intraperitoneal; i.h. intrahippocampal; i.v. intravenous; s.c. subcutaneous; bid= twice a day; w= week; P= postnatal day.

Table S2. Natural cannabinoids tested in preclinical seizure models.

Model	Cannabinoid	Vehicle/dose	Effect	Reference
**Hyperthermia induced seizures**<br>Body temperature increased 0.5 °C/2 min<br>(until first clonic convulsion-loss of posture or until 42.5 °C was	**Cannabichromene (CBC, >95%)**<br>**5-fluoro-CBC**<br>**Cannabichromenic acid (CBCA, >99%)**<br>**Cannabichromevarinic acid**	**(synthesized or donated)**<br>veh: ethanol/Tween-80/saline (1:1:18)<br>Experimental time points (i.p.):<br>45 min, 5-fluoro-CBC;<br>30 min, CBC and CBCA;	100 mg/kg CBC, 5-fluoro-CBC, CBCA, CBCVA<br>Anti-seizure effect against hyperthermia induced seizures<br>↑ GTCS temperature	**[149]**

reached)<br><br>P14-16 male and female Scn1a+/− mice	**(CBCVA, >95%)**	15 min, CBCVA	threshold	
**Hyperthermia induced seizures**<br>Body temperature increased 0.5 °C/2 min<br>(until first clonic convulsion-loss of posture or until 42.5 °C was reached)<br><br>**MES**<br>Corneal electrodes<br>60 Hz, 0.4 s, 0.5-ms/ rectangular pulse width/starting 50 mA<br><br>P14-16 male and female Scn1a+/− mice	**Cannabigerolic acid (CBGA) Cannabigerovarinic acid (CBGVA) Cannabidivarinic acid (CBDVA)**	**CBGA (THC Pharm GmbH and Epichem) CBGVA/CBDVA (synthesized)**<br>veh: vegetable oil<br>10, 30, 100 mg/kg<br>Experimental time points (i.p.):<br>45 min-CBGA<br>15 min-CBDVA and CBGVA	100 mg/kg CBGA, CBGVA, CBDVA<br>Anti-seizure effect against hyperthermia induced seizures<br>↑ GTCS temperature threshold<br><br>30 mg/kg CBGA<br>Anti-seizure effect against MES	**[150]**
**Hyperthermia induced seizures**<br>Body temperature increased 0.5 °C/2 min<br>(until first clonic convulsion-loss of posture or until 42.5 °C was reached)<br><br>P14-16 male and female Scn1aRX/+ mice	**Cannabidiolic acid (CBDA)**	**CBDA (THC Pharm GmbH)**<br>veh: ethanol/Tween 80/saline (1:1:18)<br>veh: vegetable oil (95% canola oil, 5% sunflower oil)<br>1, 10, 30 mg/kg i.p.	10 and 30 mg/kg CBDA<br>Anti-seizure effect against hyperthermia induced seizures<br>↑ GTCS temperature threshold	**[152]**
**MES**<br>Corneal electrodes<br>0.2 s, 60 Hz, 150 mA stimulus<br><br>Male Sprague-Dawley rats	**Chylobinoid Mg-CBDA**	**Chylobinoid (Synthonics, Inc.)**<br>74.5% CBDA, 4.7% CBD, 1.9% THCa, 0.3% Δ9-THC, 3.43% Mg<br>**Mg-CBDA (Synthonics, Inc.)**	Protection against MES<br>Chylobinoid (ED50= 76.7 mg/kg)<br>Mg-CBDa (ED50= 115.4 mg/kg)	**[154]**

		92.8% CBDA, 0.2% Δ9-THC, 2.76% Mg<br>veh: ethanol/Kolliphor® EL/0.9% saline (1:1:18)<br>i.p. 1 hour prior to stimulation		
**Refractory epileptic seizures**<br>Dogs (3 females, 11 males)<br>median age= 6.3 years<br>median body weight= 31.3 kg	**CBD/CBDA-rich hemp extract oil**	**CBD/CBDA-rich hemp extract oil**<br>veh: sesame seed oil<br>30 mg/mL CBD; 31 mg/mL CBDA; 1.2 mg/mL Δ9-THC; 1.3 mg/mL THCa; 1 mg/mL CBC; 1.2 mg/mL CBCA<br>2 mg/kg, oral/bid/12 w	CBD/CBDA-rich hemp extract<br>🡻 epileptic seizure frequency<br>when used concurrently with other ASMs	**[155]**
**Multi-electrode array (MEA) recordings**<br>Rat hippocampal brain slices<br>Addition: 100 mM 4-aminopyridine (4-AP)<br>Omission: MgSO4.7H2O<br><br>**MES**<br>30 mA/100 Hz/200 ms<br>earlap clamps, CD-1 mice<br><br>**Audiogenic seizures**<br>Bell (110-120 dB) was activated until occurrence of a tonic audiogenic seizure or for a maximum of 60 s<br>DBA/2 mice<br><br>**PTZ induced seizures**<br>85 mg/kg i.p./ rat	**Cannabidivarin (CBDV)**	**CBDV (GW Pharmaceuticals)**<br>***In vitro***<br>1, 10, 100 mM<br>30 min/each<br><br>***In vivo***<br>veh: etanol/Cremophor/saline (2:1:17)<br>50, 100 or 200 mg/kg<br>Experimental time point (i.p.): 1 h or 30 min	*In vitro*<br>100 mM, with 4-AP<br>🡻amplitude of epileptiform LFPs<br><br>≥10 mM, Mg2+-free conditions<br>🡻amplitude and duration of epileptiform LFPs<br><br>*In vivo*<br>Anti-seizure effects<br>MES: 100 mg/kg<br>Audiogenic seizures: 50 mg/kg<br>PTZ-induced seizures: 100 mg/kg<br><br>200 mg/kg had no effect against pilocarpine induced seizures	**[151]**

**Pilocarpine induced seizures**<br>380 mg·kg-1 i.p./ rat<br><br>Male Wistar Kyoto rats				
**PTZ induced seizures**<br>85 mg/kg i.p./ rat<br><br>**Pilocarpine induced seizures**<br>380 mg·kg-1 i.p./ rat<br><br>**Audiogenic seizures**<br>60 s (110–120 dB)<br>DBA/2 mice<br><br>Male Wistar Kyoto rats	**CBDV extracts**	**CBDV extracts (GW Pharmaceuticals)**<br>veh: etanol/Cremophor EL/saline (2:1:17)<br>Unmodified CBDV extract<br>(47.4% CBDV, 13.9% CBD, 1% Δ9-THC, 2.5% Δ9-THCV)<br>Modified CBDV extract<br>(57.8% CBDV, 13.7% CBD)<br>50-422 mg/kg<br>Experimental time point (i.p.): 1 h	Anti-seizure effects PTZ induced seizures: ≥100 mg/kg Audiogenic seizures: ≥87 mg/kg<br><br>≥100 mg/kg suppressed pilocarpine induced seizures	**[157]**
**PTZ induced seizures**<br>95 mg/kg i.p./ rat<br><br>Wistar-Kyoto rats	**CBDV**	**CBDV (GW Pharmaceuticals)**<br>veh: 20% solutol/0:9% NaCl<br>400 mg/kg oral gavage<br>Experimental time point: 3.5 h	400 mg/kg<br>↓ seizure severity<br>↑ latency to the first seizure onset	**[156]**
**PTZ induced seizures**<br>100 mg/kg s.c.<br><br>**DMCM induced seizures**<br>(methyl-6,7-dimethoxy-4-ethyl-beta-carboline-3-carboxylate)<br>(i.p.) dose 600 ug/kg/P10 rats dose 900 ug/kg/P20 rats<br><br>**KA induced seizures**	**Synthetic CBDV**	**Synthetic CBDV (Cayman Chemical)**<br>veh: etanol/Kolliphor/0.9% saline (2:1:17)<br>50, 100, 200 mg/kg i.p.<br>Experimental time point: 1h	PTZ induced seizures<br>100 and 200 mg/kg (P10)<br>↓seizure severity<br>200 mg/kg (P20)<br>↓seizure score<br>↑ latency to seizure onset<br><br>MES<br>(P20) protected against tonic hindlimb extension<br><br>DMCM induced	**[158]**

(i.p.) dose 4 mg/kg/P10 rats dose 7 mg/kg/P20 rats<br><br>**MES**<br>Transorbital (P10) stimulation<br>60Hz sinusoidal train/pulses 110mA/200ms<br>Transcorneal (P20) stimulation<br>200Hz train/0.9ms-wide square pulses/50mA/300ms<br><br>P10 and P20, male and female Sprague-Dawley rats			seizures<br>200 mg/kg, P20 rats<br>↓seizure severity<br><br>KA induced seizures (P10, P20) not changes in seizure severity nor latency to seizure onset	
**PTZ induced seizures**<br>50 μL-20 mM/ final concentration: 5 mM<br><br>**Dravet syndrome (DS) scn1Lab−/− mutant model**<br><br>Scn1Lab−/− and wild-type zebrafish	**Cannabinol (CBN) Linalool (LN)**	**CBN and LN (Sigma)**<br>0.25- 0.6- 1- 4 μM<br>veh: 0.05% DMSO<br>24 h pre-exposure	CBN and LN not reduce zebrafish PTZ induced seizures<br><br>CBN and LN ↓seizures in the DS model	**[160]**
**6-Hz corneal stimulation test** (44 mA)<br>once a day/ 5 days<br>current intensity 44 mA;<br>pulse width, 0.2 ms;<br>duration, 3 s;<br>frequency, 6-Hz;<br>corneal electrodes<br><br>Male CD-1 mice	**Oil hemp extracts**	**Oil hemp extracts (prepared)**<br>**hemp oil 1** (K1; without volatile compounds)<br>**hemp oil 2** (K2; extract rich in terpenes)<br>veh: olive oil<br>25 mg/kg/day/oral gavage<br>Experimental time point: 1 h	↓ %animals experiencing GTCS<br>K1 (12.5%)<br>K2 (25.0%)	**[161]**

CBD, cannabidiol; THCa, tetrahydrocannabinolic acid; Mg, magnesium; Δ9-THC, Δ9-tetrahydrocannabinol; Δ9-THCV, Δ9-tetrahydrocannabivarin; ASMs, anti-seizure medications; ED_{50} = median effective dose; MES, maximum electroshock seizures; PTZ, pentylentetrazole; KA, kainic acid; GTCS, generalized tonic-clonic

seizures; LFPs, local field potentials; veh=vehicle; i.p. intraperitoneal; s.c. subcutaneous; bid= twice a day; w= week; P= postnatal day.
